# Stress Analysis in Single-Lap Adhesive Joints: Comparison of Unreinforced, Reinforced and Prestressed Configurations Assembled with Brittle Structural Adhesives

**DOI:** 10.3390/ma18184224

**Published:** 2025-09-09

**Authors:** Francesco Marchione

**Affiliations:** Dipartimento di Ingegneria Civile, Edile e Architettura (DICEA), Università Politecnica Delle Marche, 60131 Ancona, Italy; f.marchione@pm.univpm.it

**Keywords:** multi-layered adhesives, single-lap adhesive joints, hybrid bonded joints, layered adhesive

## Abstract

Adhesive joints provide an effective and lightweight solution for the assembly of structures and offer both mechanical and operational advantages over conventional mechanical fastening systems. In this study, a new single-layer adhesive joint is investigated in which a thin, pretensioned textile reinforcement is inserted into the adhesive layer. In the first part, a simplified analytical model is proposed to describe the distribution of axial stresses in the adhesives and the reinforcement as well as the shear stresses in the adhesive layer. In the second part, the effects of geometric, mechanical and loading variables are investigated in a parametric analysis, focussing on the role of the initial pre-compression on the tensile response of the joint. The third part of this study compares the theoretical results with experimental data obtained with static tests on specimens made of unreinforced GFRP and epoxy resin. The results show small deviations (3–8%) between model and test. Finally, a simplified method for estimating the load-bearing capacity of brittle joints, both conventional and reinforced, is proposed. It is shown how the introduction of reinforcement and prestressing can modulate the stiffness and improve the stability of the joint without significantly affecting the load-bearing capacity.

## 1. Introduction

Adhesive joints have developed considerably in scientific research in recent years [1]. Their widespread and increasing use is mainly due to the advantages they offer over conventional joining techniques: lower weight, more even stress distribution, and easier assembly compared to welded and bolted joints [2,3]. An important advantage of adhesive joints, compared to bolted or riveted joints, is the elimination of the hole effect, which causes local stress concentrations (stress concentration factor, SCF) in the connected materials, allowing for a more uniform stress distribution and higher structural efficiency of the joint. In addition, adhesive and combination joints, such as bond-riveted, exhibit high shear strength and good energy absorption capacity [4].

The areas of application range from the automotive industry [5,6] to aerospace [7,8] and construction [9,10,11]. In addition to their widespread use in structural applications, adhesives have become the subject of extensive non-destructive evaluation studies, such as in refs. [12,13]. Techniques such as ultrasonic testing are increasingly applied to assess the quality and integrity of adhesive joints, including those manufactured with additive manufacturing technologies. These methods provide valuable insights into defects, bonding quality and the overall performance of structural adhesives, supporting both research and industrial applications, as in the works of Cavalcanti et al. [14,15].

Since the early 1940s, numerous studies have been conducted to determine the knowledge of the stress state and apply improvements to modify the failure methods [16,17,18]. These investigations have progressively addressed aspects such as the influence of adhesive type, joint geometry, loading conditions and surface preparation on stress distribution and joint strength. Over the decades, both experimental and analytical approaches have contributed to a deeper understanding of failure mechanisms, leading to optimized designs and more reliable structural applications.

In this sense, closed-form solutions have always proved to be a valuable tool. The best-known solutions are derived from linear elastic problems, which allow useful approximations to the solution [19]. In fact, non-linear assumptions inevitably lead to an increase in the assumptions of the problem, which requires more elaborate solutions that can often only be performed with the help of powerful computers. Pioneering analytical models for bonded joints were developed as early as 1938 by Volkersen [18], for single-lap joints where only the adhesive was considered to respond to shear and the adherends to normal stresses. Later developments were recorded with the studies by Goland and Reissner [20] in 1944, which introduced normal forces into the adherends. It was not until the studies of Hart-Smith [21] that plasticity in adhesives began to be discussed. Subsequently, thanks in part to the advent of finite element solvers, numerous studies have been conducted to deepen our understanding of the mechanisms of adhesion, most notably the efforts of Adams et al. [22,23,24], pioneers in the field of FEM applied to adhesive technology.

Despite numerous studies, the design of adhesive bonding is still very laborious, both because of the highly non-linear behaviour of adhesives [25,26] and because of their durability, which is still poorly understood due to the unknown behaviour in their exposure to environmental agents [27,28].

In the recent literature, there are many studies investigating methods to reduce stress peaks in the adhesive region, which are usually responsible for the failure of the adhesive systems. Marchione [29] compared a classical bonded joint with a modified version using tapered adherends to reduce stress peaks in the adhesive layer. Using a 3D FEM analysis of single-lap steel joints with epoxy adhesive, he found that a taper angle of 5° reduces normal stresses by up to 30%, while larger angles bring no further advantage. The tangential stresses, on the other hand, increase with the angle. Tapering is therefore useful to reduce normal stresses, the main cause of damage. Deng and Lee [30] analysed several conventional joining techniques applied to structural reinforcement with CFRP plates. In particular, they studied the use of splices (spew fillets) and end tapers (end tapers) to reduce stress concentrations in the adhesive layer of reinforced beams. By means of finite element (FE) analyses, they compared eight different configurations, revealing that the combination of an internal spew fillet and a triangular fillet is the most effective in decreasing inter-facet stresses. The results also confirm, in line with previous studies, that the size of the fillets affects the deformations near the end of the plate, although the effect is less than with overlap joints. In [31], Marchione numerically analysed single-lap joints between GFRP adherends and evaluated the effect of hollow adherends in the bonding area to reduce stress peaks in the adhesive layer. Different geometric configurations and two adhesive types (epoxy and polyurethane) were considered. The results show that the symmetrical use of hollow profiles in both adherends effectively reduces stress peaks, especially for higher cavity heights, while offering an advantage in terms of the overall weight of the joint. Shishesaz and Reza [32] investigated the effect of the viscoelastic behaviour of polymer adhesives on the distribution of shear stresses in double-lap adhesive joints subjected to shear stress. Using a three-layer film with elastic adhesives and viscoelastic adhesive, they solved the equations in the Laplace domain. The results show that the initial shear stress in the adhesive gradually decreases under gradual loading until it stabilises after about 1000 s.

Vallée et al. [33] investigated experimentally and numerically the effectiveness of three methods of stress reduction (adhesive bevels, adhesion bevels and gradient adhesives) on adhesive joints with brittle FRP and wood adherends. The experimental results showed a negligible increase in strength, while the numerical analyses showed that although the stress peaks were reduced, they were distributed over a larger volume. The use of a probabilistic strength prediction model made it possible to explain these effects and showed that for joints with brittle adhesives, a reduction in stress does not automatically guarantee an increase in strength.

An interesting study was carried out by Xiong et al. [34]. The authors conducted both an analytical and a finite element method (FEM) study to analyse the overall strength and interfacial stress distribution in single-lap adhesive joints containing an inclusion embedded in the adhesive layer. They developed a stress analysis model for these joints, including the inclusion layer, and investigated how the thickness and stiffness of the inclusion affects the mechanical strength of the joints. The results show that the strength of the joint strongly depends on the geometrical and mechanical properties of the inclusion and increases with the thickness and stiffness of the inclusion. On the other hand, if the stiffness of the inclusion is lower than that of the adhesive, the strength of the joint deteriorates due to the presence of the inclusion.

Finally, the FEM analysis made it possible to visualise the stress distribution and the failure mechanisms associated with the inclusions and confirmed the results of the analytical method.

The state of the art in the field of adhesive joints is very extensive and widely studied. There are numerous studies analysing various aspects of strength and stress distribution in single-lap joints. To date, however, there are no specific solutions in the literature for the introduction of an inclusion in the adhesive layer, which is pretensioned before bonding. This particular configuration, which could significantly influence the mechanical behaviour of the joint, has not been studied in detail to date.

The present study focuses precisely on this innovative aspect and analyses the stress state of a single-lap joint in both the classic and the prestressed inclusion-reinforced configuration. The investigation is carried out parametrically, taking into account the variable effects of external loads, the degree of prestressing of the inclusion, different joint geometries and mechanical parameters of the materials involved. In this way, this study aims to understand in detail how these variables affect the stress distribution and the overall strength of the joint.

In addition, a simplified and fast calculation method for estimating the stress distribution and overall strength of the joint is developed, applicable in particular to configurations with rigid epoxy adhesives. This expeditious method, based on the results of parametric analysis, is a useful tool for the design and evaluation of reinforced adhesive joints, offering a practical and reliable approach for engineers and designers.

## 2. Theoretical Model

This section presents the analysis of the mechanical model of the single-lap adhesive joint in both the classical configuration and reinforced by prestressing applied in the adhesive layer. This section shows the solutions obtained in analytical form for the distribution of stresses within the adhesive region and for the distribution of axial stresses within the adherends. The simplifications of the model and the assumptions made for the calculation of the stresses are as follows:

i All elements are homogeneous, elastic, linear and isotropic;
ii The outer adherends are made of the same material;
iii The tensile load is applied uniformly at the ends of the adherends;
iv Prestressing is considered constant over time;
v A plane strain condition is considered.

In the analysis conducted, it was assumed, as a simplifying assumption, that the pretension force in the adhesive joint remains constant over time. Although this assumption does not take into account the viscoelastic relaxation effects typical of polymeric materials, it allows us to reduce the complexity of the model and focus attention on the geometric and mechanical aspects under study.

### 2.1. Stress Distribution in URM Single-Lap Adhesive Joints (UR-SLJ)

**Figure 1:**
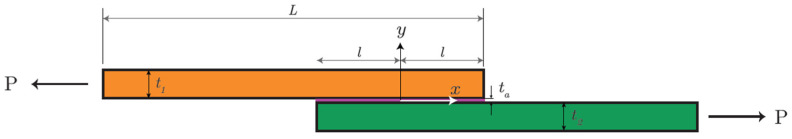
Single-lap adhesive joint scheme (URM configuration).

**Figure 2:**
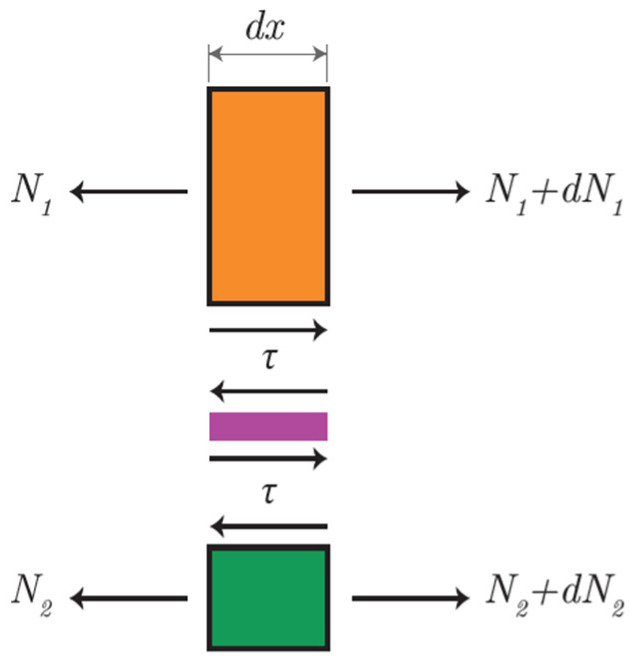
Free body diagram (URM configuration).

Figure 1 illustrates the diagram of the single-lap adhesive joint in the classic configuration. Figure 2 shows the free body pattern for the joint considered. The analysis formulation is similar to that conducted by Volkersen in ref. [18]. In the following figures, the subscript indicates the load resulting from the external constraint reaction.

The longitudinal equilibrium yields

(1) $$\frac{dN_{1}}{dx}+\tau =0$$

(2) $$\frac{dN_{2}}{dx}-\tau =0$$

The relative displacement between adherends is given by

(3) $$s=u_{2}-u_{1}$$

Taking Timoshenko’s kinematic assumptions as valid and considering the adhesive as an elastic ground in which tension and shear deformation are directly proportional for a quantity k, the following relationship can be obtained:

(4) $$\tau =k_{s}s$$

where

(5) $$k_{s}=\frac{G_{a}}{t_{a}}$$

where $G_{a}$ represents the adhesive shear modulus and t_a_ the adhesive thickness. Combining Equations (3)–(5) yields the following:

(6) $$\tau \left(x\right)=\frac{G_{a}}{t_{a}}\left(u_{2}-u_{1}\right)$$

Considering the following constitutive relationships (axial deformations) for the adherents, the following can be assumed:

(7) $$\varepsilon_{i}=\frac{du_{i}}{dx}=\frac{N_{i}}{E_{i}t_{i}}$$

Deriving Equation (6) and combining it with Equation (7), we obtain the following differential equation:

(8) $$\frac{d^{2}N_{1}}{dx^{2}}-\alpha^{2}N_{1}=-\frac{{F\;G}_{a}}{E_{2}t_{2}t_{a}}$$

where

(9) $$\alpha =k_{s}\left(\frac{1}{E_{1}t_{1}}+\frac{2}{E_{2}t_{2}}\right)$$

Solving Equation (8) with the following BCs,

(10) $$N_{1}=0;\;x=l$$

(11) $$N_{1}=\frac{F}{2};\;x=-l$$

The following solutions are obtained for the axial stresses in the adherends and the distribution of shear stresses in the adhesive layer:

(12) $$\tau \left(x\right)=\alpha \frac{F}{4}\left(\frac{e^{\alpha x}+e^{-\alpha x}}{e^{\alpha l}-e^{-\alpha l}}-\beta \frac{e^{\alpha x}-e^{-\alpha x}}{e^{\alpha l}+e^{-\alpha l}}\right)$$

(13) $$N_{2}\left(x\right)=F\left[1+\frac{e^{\alpha x}-e^{-\alpha x}}{2\left(e^{\alpha l}-e^{-\alpha l}\right)}-\frac{E_{2}t_{2}-2E_{1}t_{1}}{{2E}_{2}t_{2}+4E_{1}t_{1}}\frac{e^{\alpha x}+e^{-\alpha x}}{e^{\alpha l}+e^{-\alpha l}}-\frac{2E_{1}t_{1}}{E_{2}t_{2}+2E_{1}t_{1}}\right]$$

(14) $$N_{1}\left(x\right)=\frac{F}{2}\left[-\frac{1}{4}\frac{e^{\alpha x}-e^{-\alpha x}}{e^{\alpha l}-e^{-\alpha l}}+\frac{E_{2}t_{2}-2E_{1}t_{1}}{{2E}_{2}t_{2}+4E_{1}t_{1}}\frac{e^{\alpha x}+e^{-\alpha x}}{e^{\alpha l}+e^{-\alpha l}}+\frac{2E_{1}t_{1}}{E_{2}t_{2}+2E_{1}t_{1}}\right]$$

where

(15) $$\beta =\frac{E_{2}t_{2}-2E_{1}t_{1}}{E_{2}t_{2}+{2E}_{1}t_{1}}$$

### 2.2. Stress Distribution in RM Single-Lap Adhesive Joints (RM-SLJ)

**Figure 3:**
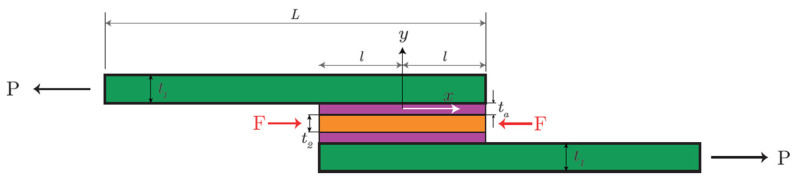
Single-lap adhesive joint scheme (RM configuration).

**Figure 4:**
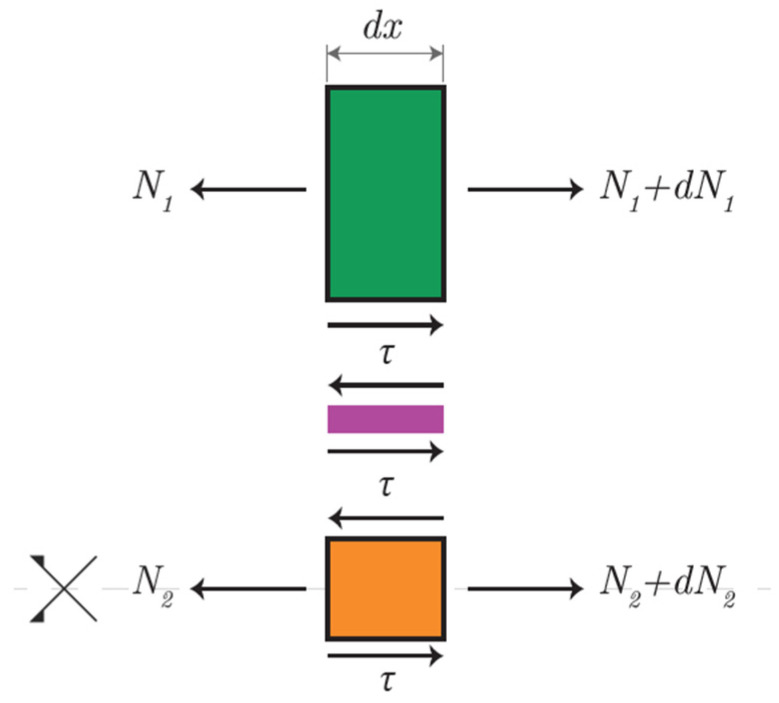
Free body diagram (RM configuration).

Figure 3 shows a schematic for the single-lap joint with reinforced adhesive layer with pretensioned insert. Figure 4 shows a free body diagram for the reinforced single-lap joint.

The longitudinal equilibrium yields

(16) $$\frac{dN_{1}}{dx}+\tau =0$$

(17) $$\frac{dN_{2}}{dx}=0$$

Taking the assumptions illustrated for the unreinforced configuration as valid and similarly operating the same algebraic substitutions, the following differential equation is obtained in terms of N_1_.

(18) $$\frac{d^{2}N_{1}}{dx^{2}}-\left[\frac{G_{a}}{t_{a}}\left(\frac{1}{E_{1}t_{1}}+\frac{1}{E_{2}t_{2}}\right)\right]N_{1}=-\frac{G_{a}}{t_{a}}\frac{\left(P-F\right)}{E_{2}t_{2}}$$

Solving Equation (18) with the following BCs,

(19) $$N_{1}=0;\;x=l$$

(20) $$N_{1}=P-F;\;x=-l$$

For global equilibrium we have

(21) $$P=N_{1}+N_{2}-F$$

The following solutions are obtained for the axial stresses in the adhesives and the distribution of shear stresses in the adhesive layer:

(22) $$N_{1}\left(x\right)=\left(P-F\right)\frac{\sinh\left(\sqrt{\frac{G_{a}}{t_{a}}\left(\frac{1}{E_{1}t_{1}}+\frac{1}{E_{2}t_{2}}\right)}\left(l-x\right)\right)}{\sinh\left(2l\sqrt{\frac{G_{a}}{t_{a}}\left(\frac{1}{E_{1}t_{1}}+\frac{1}{E_{2}t_{2}}\right)}\right)}+\frac{P-F}{\frac{E_{2}t_{2}}{E_{1}t_{1}}+1}\left[1-\frac{\cosh\left(x\sqrt{\frac{G_{a}}{t_{a}}\left(\frac{1}{E_{1}t_{1}}+\frac{1}{E_{2}t_{2}}\right)}\right)}{\cosh\left(l\sqrt{\frac{G_{a}}{t_{a}}\left(\frac{1}{E_{1}t_{1}}+\frac{1}{E_{2}t_{2}}\right)}\right)}\right]$$

(23) $$N_{2}\left(x\right)=\left(P-F\right)\left[1-\frac{\sinh\left(\sqrt{\frac{G_{a}}{t_{a}}\left(\frac{1}{E_{1}t_{1}}+\frac{1}{E_{2}t_{2}}\right)}\left(l-x\right)\right)}{\sinh\left(2l\sqrt{\frac{G_{a}}{t_{a}}\left(\frac{1}{E_{1}t_{1}}+\frac{1}{E_{2}t_{2}}\right)}\right)}\right]-\frac{P-F}{\frac{E_{2}t_{2}}{E_{1}t_{1}}+1}\left[1-\frac{\cosh\left(x\sqrt{\frac{G_{a}}{t_{a}}\left(\frac{1}{E_{1}t_{1}}+\frac{1}{E_{2}t_{2}}\right)}\right)}{\cosh\left(l\sqrt{\frac{G_{a}}{t_{a}}\left(\frac{1}{E_{1}t_{1}}+\frac{1}{E_{2}t_{2}}\right)}\right)}\right]$$

(24) $$\tau \left(x\right)=\left(P-F\right)\frac{\sqrt{\frac{G_{a}}{t_{a}}\left(\frac{1}{E_{1}t_{1}}+\frac{1}{E_{2}t_{2}}\right)}\cosh\left(\sqrt{\frac{G_{a}}{t_{a}}\left(\frac{1}{E_{1}t_{1}}+\frac{1}{E_{2}t_{2}}\right)}(l-x)\right)}{\sinh\left(2l\sqrt{\frac{G_{a}}{t_{a}}\left(\frac{1}{E_{1}t_{1}}+\frac{1}{E_{2}t_{2}}\right)}\right)}-\frac{P-F}{\frac{E_{2}t_{2}}{E_{1}t_{1}}+1}\frac{\sqrt{\frac{G_{a}}{t_{a}}\left(\frac{1}{E_{1}t_{1}}+\frac{1}{E_{2}t_{2}}\right)}\sinh\left(\sqrt{\frac{G_{a}}{t_{a}}\left(\frac{1}{E_{1}t_{1}}+\frac{1}{E_{2}t_{2}}\right)}x\right)}{\cosh\left(l\sqrt{\frac{G_{a}}{t_{a}}\left(\frac{1}{E_{1}t_{1}}+\frac{1}{E_{2}t_{2}}\right)}\right)}$$

## 3. Parametric Study

A systematic co-parametric study was carried out to understand the influence of the main mechanical parameters on the behaviour of the prestressed adhesive joint in a single layer configuration. The variables considered include the thickness of the adhesive, the elastic moduli of the substrates, the stiffness of the adhesive and the amount of load applied during operation. Table 1 shows the mechanical and physical parameters used for analytical analysis—in cases not subject to parametric analysis.

The thicknesses of the bonded elements (up to 5 mm) and of the structural adhesive layer (2 mm) were selected with civil engineering applications in mind, such as window and door frames or other structural joints in building components. These values are representative of typical practice in these contexts, providing adequate strength and manufacturability while remaining consistent with industrial applications.

**Figure 5:**
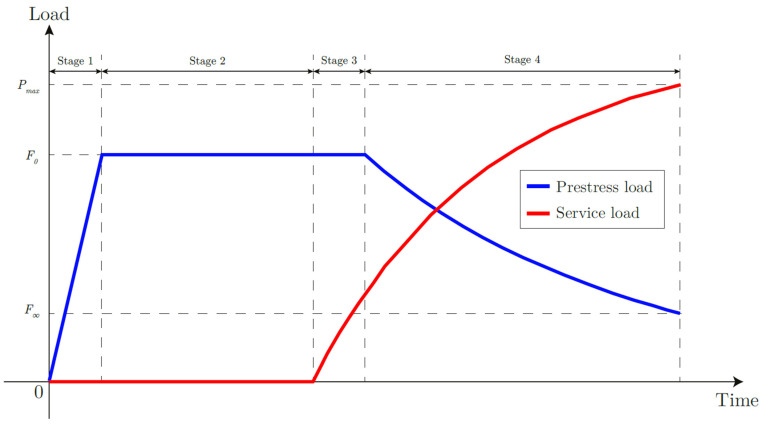
Development of external loads and applied prestress over time.

In Figure 5, the markers F and P indicate different types of loads: F corresponds to the external prestress applied to the additional adhesive layer, while P represents the external load applied to the adherend. The symbol ∞ in P∞ denotes the load value after the pretension has been fully applied and the system has reached equilibrium, highlighting the asymptotic or steady-state condition of the applied load.

The analysis was carried out taking into account the temporal evolution of the forces acting in the joint—as shown in Figure 5—distinguishing three main phases:

- The application of initial prestressing (Stage 1);
- The adhesive cure period where the prestressing remains constant (Stage 2);
- The subsequent commissioning phase with the introduction of static external traction forces until the joint fails (Stages 3–4).

The proposed analysis is limited to a static evaluation, referring to the phase in which the pretension and external forces can be considered constant. This approach makes it possible to validate the model and to highlight the effects of pretensioning in the operational phase. Analyses relating to pressure losses and their temporal evolution will be the subject of future studies.

The results clearly show how the relative stiffness between substrate and adhesive influences both the stress distribution in the bond and the stability of the preload over time. The introduction of the external load during the prestressing phase activates a superposition of mechanical effects that can jeopardise the integrity of the joint, especially for adhesives with low residual viscosity or low thickness. This study is limited to analysing the phases in which only the prestressing and simultaneously the prestressing and the external load are present, for fixed values corresponding to the elastic field of the joint.

### 3.1. Stages 1–2: Effect of the Sole Prestress

**Figure 6:**
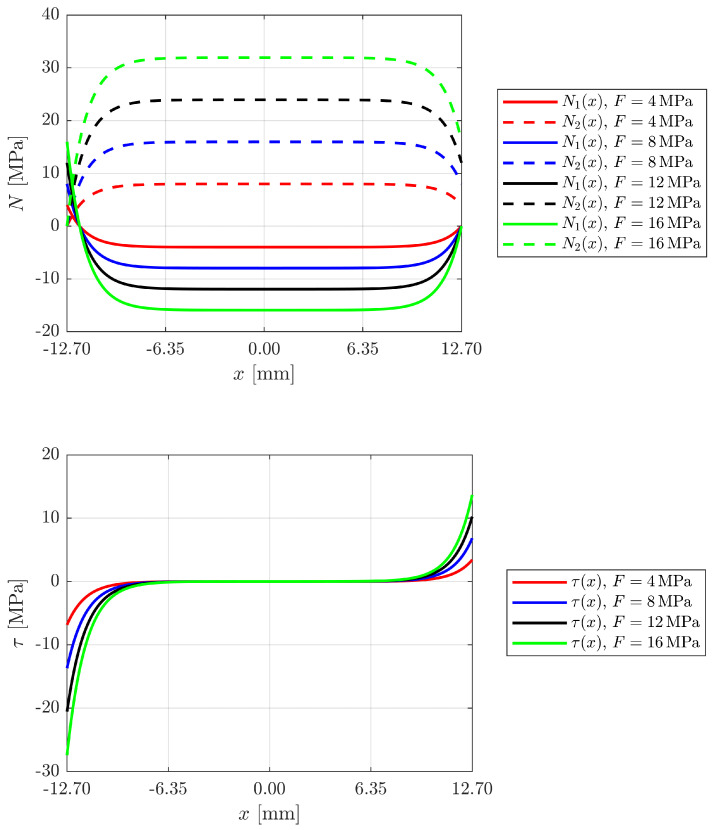
Development of axial stresses in the adherends and shear stresses in the adhesive layer at varying prestressing (zero external loads).

Figure 6 illustrates the development of axial stresses in the adhesives and shear stresses in the adhesive layer as the prestress applied to the reinforcement changes, in the absence of external loads.

This behaviour illustrates the different mechanical reaction of the connection components under preload. Increasing the prestressing leads to an almost linear increase in the axial stresses in the substrates and the reinforcement, which are distributed relatively evenly in the central area of the joint due to the continuity of the material. However, this distribution is strongly disturbed near the ends of the bonded area, where a rapid stress drop is observed: this is due to mechanical discontinuity effects and differential rotation caused by the eccentrically loaded configuration of the single layer joint.

In contrast, the adhesive layer shows the opposite behaviour: the central part of the layer remains essentially “unloaded”, as the axial stress is largely diverted to the stiffer substrates. At the ends, however, there are very high stress concentrations (up to 20–30 MPa) caused by a combination of strain gradients, different stiffnesses of the materials and, in some cases, peeling effects. This localised stress peak is the critical point for the integrity of the joint as it can cause delamination or premature damage, especially with brittle or low-strength adhesives.

### 3.2. Stages 3–4: Combined Effect of Constant Pretension and Increasing External Loads

**Figure 7:**
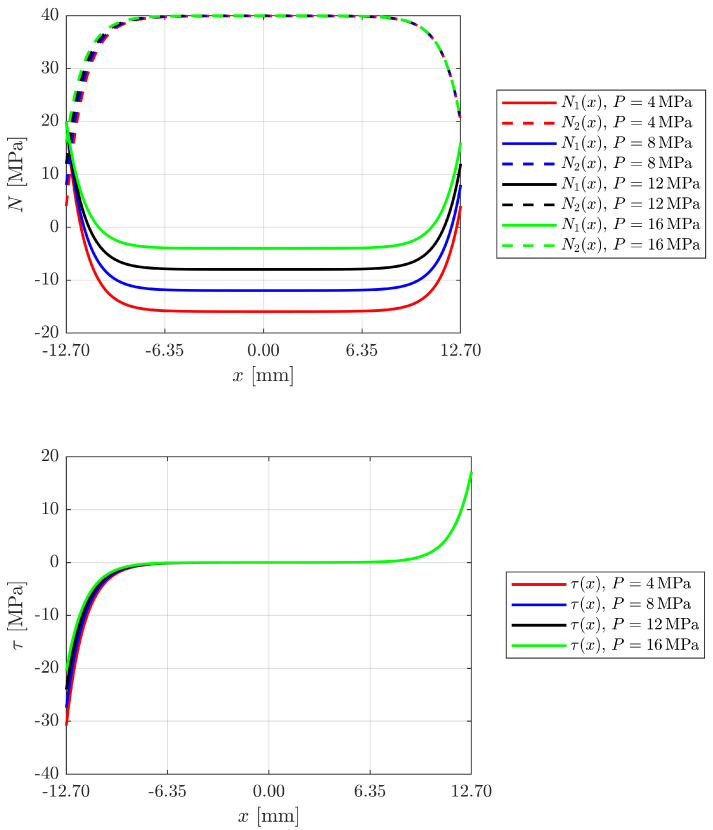
Development of axial stresses in the adhesives and shear stresses in the adhesive layer at varying external loads (constant prestressing).

Figure 7 illustrates the development of prestress under varying external loads, where the pretension value can be considered constant over time.

The increase in the external load leads to a proportional increase in the axial stresses in the outer adhesive layer in joints with a constantly prestressed reinforcement. In contrast, the reinforcement layer is mechanically less stressed by the change in the external load: the axial stresses in it remain almost unchanged, which shows that the resistant component of the reinforcement remains dominated by the original prestress and does not participate significantly in the redistribution of the stresses induced by the external load.

In the bonded layer, the usual concentration of localised stresses can be observed at the ends of the bonded area. On the left side of the joint, however, a counterintuitive phenomenon occurs: as the external load increases, the value of the peak adhesive stress decreases significantly. For example, it decreases from about 30 MPa at an external load of 4 MPa to about 20 MPa at an external load of 16 MPa. This behaviour can be interpreted in the light of the principle of superposition of mechanical effects and the local equilibrium of stresses in the joint. In fact, the adhesive initially works under dominant prestressing conditions with a strong local stress gradient. The introduction of the external load changes the stress field, partially acting in antiphase to the prestressing (depending on the point along the joint) and generating a differential relaxation of the localised stresses. This redistribution tends to reduce the local peaks at the points originally under the greatest load, while other, previously less loaded areas of the joint begin to participate in the transfer of the load.

This behaviour can be partly attributed to a combination of the different stiffness of the materials, the peeling effect at the ends and the local non-linearity created by the combination of axial loading and tangential creep. From a design perspective, these findings suggest that appropriate modulation of the preload and loading sequence can help to reduce stress concentrations and improve the durability of the bonded joint.

### 3.3. Effect of the Stiffness of the Pretensioned Reinforcement

**Figure 8:**
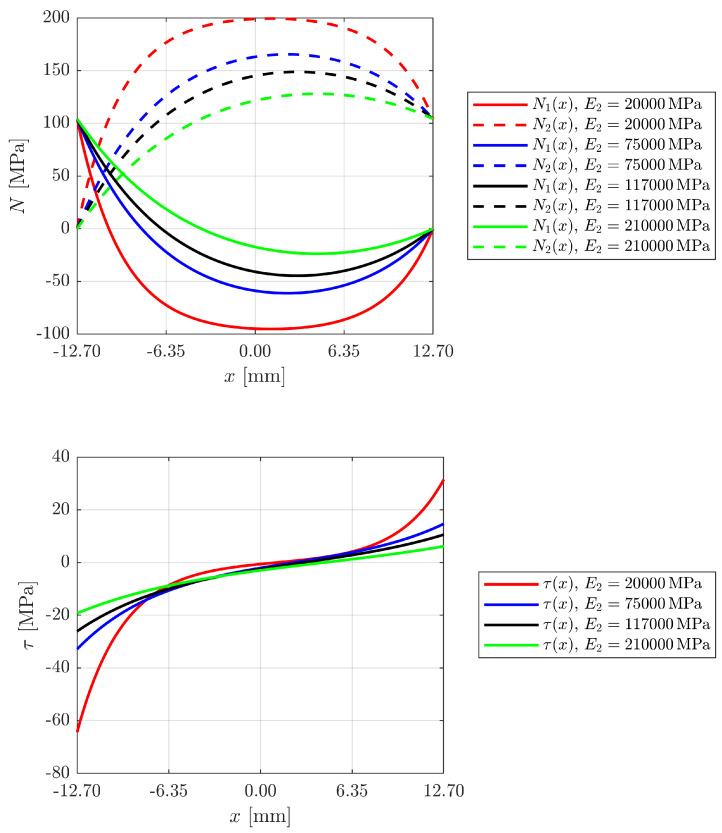
Development of axial stresses in the adhesives and shear stresses in the adhesive layer as the prestressed reinforcement material changes.

Figure 8 illustrates the stress trend as the material used as prestressing reinforcement in the adhesive layer changes.

The analysis shows how the stiffness of the reinforcement material significantly influences the stress distribution in the bonded joint. With increasing modulus of elasticity of the reinforcement—from reinforced polymeric materials (e.g., polyamide, E = 20,000 MPa) to metals with high stiffness such as steel (E = 210,000 MPa)—a progressive reduction in the axial stresses in the outer adhering layer can be observed, in favour of increased load absorption by the reinforcement. At the same time, the tangential stresses in the adhesive layer are significantly reduced as the stiffness of the reinforcement increases, from very accentuated peaks in the case of GFRP to more restrained and distributed values in the case of steel. This behaviour is consistent with the greater deformation compatibility of less stiff materials, which, however, lead to a higher load on the adhesive in the end zones. The results presented show the need for a balance between the stiffness of the reinforcement and the stress distribution, depending on the type of loading and the properties of the adhesives.

## 4. Failure Criteria

This section reports the analytical determination of ultimate load for reinforced single lap adhesive joints assembled with brittle adhesives.

Since the failure of the adhesive joints was experimentally manifested as adhesive, the von Mises criterion was adopted in the numerical analyses by exclusively considering the shear stresses in the adhesive layer. This choice, while simplifying the model, allows the effects of pretension and geometric configuration on the joint failure behaviour to be consistently evaluated. All theoretical assumptions made in the previous sections are considered valid, i.e.,

(i) The adhesive has a quasi-brittle mechanical behaviour;
(ii) The adhesive has linear elastic behaviour up to the shear stress;
(iii) The joint has a triangular stress/slip behaviour with sudden drop.

The adhesives considered in this study exhibit a quasi-brittle mechanical behaviour. This implies a mechanical response characterised by an initially linear phase, followed by a short non-linear trend, at the end of which failure occurs. This quasi-brittle nature makes it possible to identify a yield value, which was taken as a reference parameter in the analyses presented. In a triangular stress/slip law hypothesis with a sudden drop, the area under the curve represents exactly the fracture energy required to cause the joint to fail; this energy is denoted by G_f_. This energy can be expressed by the following equation:

(25) $$G_{f}=\frac{1}{2}\tau_{max}\delta_{f}$$

Considering the von Mises failure criterion, which has been verified by the author in previous publications, the total stress is given by the sum of the deviatoric and shear stress. Since stress components other than shear are neglected in the present analysis, the following relationship results:

(26) $$\tau_{y}\approx \frac{\sigma_{y}}{\sqrt{3}}$$

In this study, the von Mises criterion is adopted, limited to the evaluation of shear stresses. It is recognised that failure is also influenced by peel stresses and the effects of hydrostatic pressure, which are not considered here for reasons of model simplicity.

Using Equation (26) it is possible to calculate the value of the characteristic plastic shear stress of the adhesive from experimental axial tensile tests on dogbones of adhesive.

Knowing from experimental data or data provided by the manufacturer the value of the tensile yield strength of the adhesive, it is possible to obtain the expression of the maximum load, independently of the fracture energy. In this way, the failure load of the joint can be calculated directly without first determining the fracture energy, as in Ref. [35].

The ultimate load value for unreinforced single-lap joints is shown below:

(27) $$P_{max,slj}=\frac{2\sigma_{y}}{\sqrt{3}\alpha \left(\frac{e^{\alpha l}+e^{-\alpha l}}{e^{\alpha l}-e^{-\alpha l}}-\beta \frac{e^{\alpha l}-e^{-\alpha l}}{e^{\alpha l}+e^{-\alpha l}}\right)}$$

where

(28) $$\alpha =\frac{G_{a}}{t_{a}}\left(\frac{1}{E_{1}t_{1}}+\frac{1}{E_{2}t_{2}}\right)$$

and

(29) $$\beta =\frac{E_{2}t_{2}-2E_{1}t_{1}}{E_{2}t_{2}+{2E}_{1}t_{1}}$$

The ultimate load value for reinforced and prestressed single-lap joints is shown below:

(30) $$P_{max,slj}=F+\frac{\sigma_{y}\cosh\alpha l}{\sqrt{3}\alpha \left(\frac{1}{2\sinh\alpha l}-\frac{\sinh\alpha l}{1+\beta }\right)}$$

where

(31) $$\alpha^{*}=\sqrt{\frac{G_{a}}{t_{a}}\left(\frac{1}{E_{1}t_{1}}+\frac{1}{E_{2}t_{2}}\right)}$$

and

(32) $$\beta^{*}=\frac{E_{1}t_{1}}{E_{2}t_{2}}$$

## 5. Verification

In this section, the results of a special campaign carried out on adhesive joints between GFRP adhesives, assembled with epoxy adhesive, are reported and analysed. The experimental results of this campaign are summarised in Ref. [36].

### 5.1. Materials

#### 5.1.1. Adherends

In this study three different materials were used: transparent float glass manufactured by Vetromarche (Italy), GFRP pultruded profiles manufactured by Fibrolux (Germany) and nylon 6 fabrics manufactured by Lazzati Group (Italy). The properties of the materials, as reported by the manufacturers in their technical sheets, are reported in Table 2 and Table 3.

#### 5.1.2. Adhesives

In the experimental campaign described, the focus is on the rigid epoxy resin Kimi-tech EP-TX [38]. The choice of such an adhesive is due to its widespread use on the market. The mechanical properties of the adhesive, as specified by the manufacturers, are summarised in Table 4.

### 5.2. Experimental Tests

Mechanical tests were carried out to evaluate the effectiveness of the adhesive bond between the GFRP adherends and to verify the structural contribution of a nylon reinforcement placed at half the thickness of the adhesive layer. For each test configuration, three specimens were assembled and tested. The geometry of the specimens was in accordance with ISO 4587: the adhesives were 25 mm wide and 100 mm long, and the overlap length was 12.7 mm. The thickness of the adhesives was 5 mm. The thickness of the adhesive layer ranged from 2 mm for the unreinforced joints to 4 mm for the reinforced joints.

The nylon fabric reinforcement was first cleaned manually to remove impurities (e.g., dust particles), then dried and acclimatised under ambient conditions before bonding. The surfaces of the GFRP adhesives in the bonding zone were treated by manual sanding with sandpaper and then degreased with acetone and isopropyl alcohol. For the experimental tests, the thickness of the adhesive layer was controlled during the preparation of the samples using calibrated spacers in order to ensure an even distribution of the adhesive in the joint. After the adhesive had cured, the actual thickness was measured at several points on each sample using a digital micro-meter. The average thickness obtained was 4.24 mm, with a standard deviation of 0.05 mm based on three measurements per sample.

**Figure 9:**
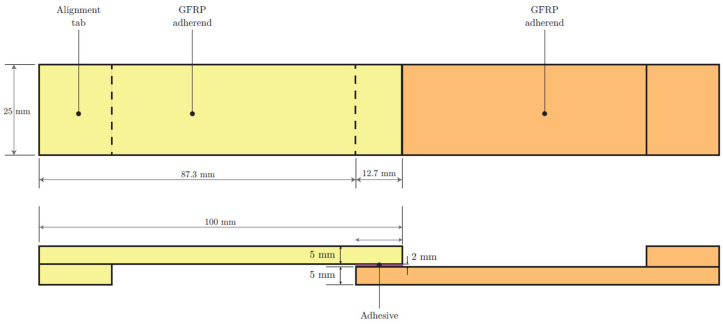
Unreinforced single-lap specimens: plan view and section.

**Figure 10:**
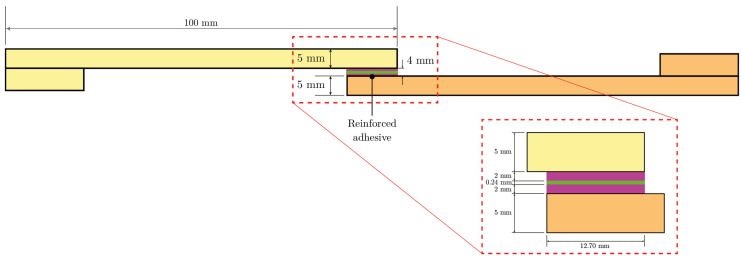
Reinforced single-lap specimens: plan view and section.

The test specimens used in the experimental campaign are illustrated in Figure 9 and Figure 10.

### 5.3. Experimental Results

**Figure 11:**
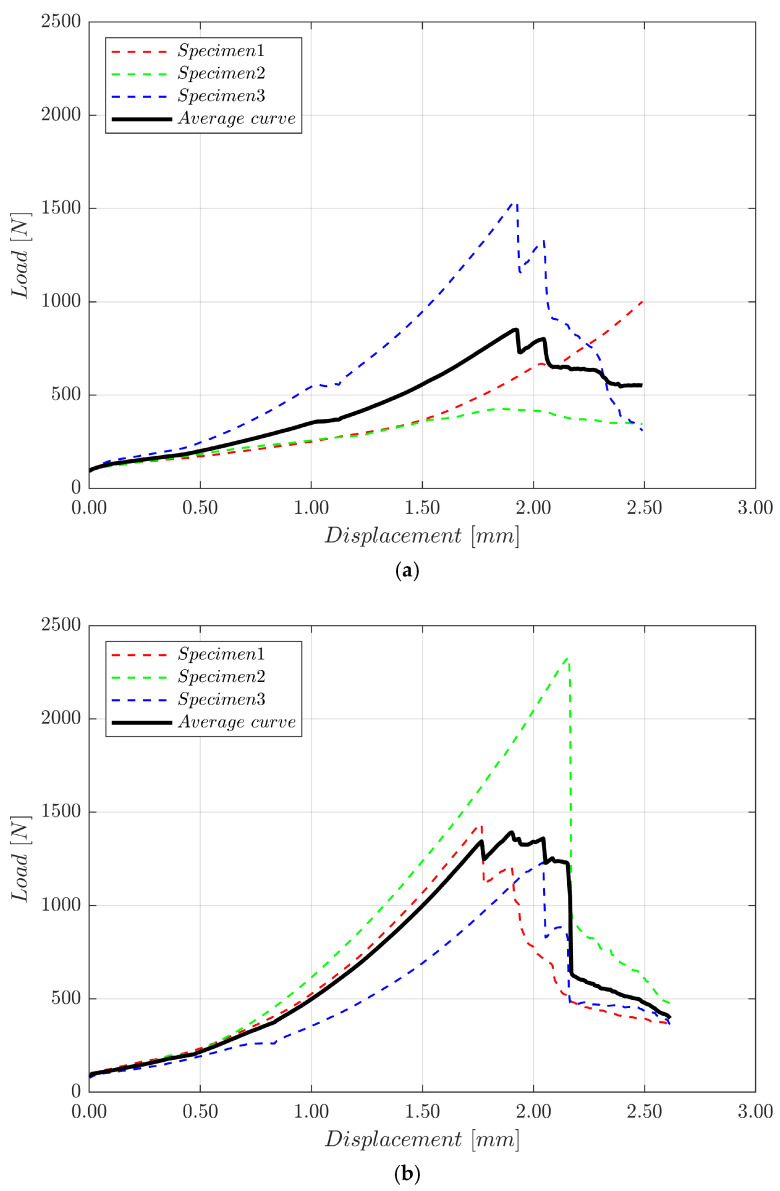
Experimental curves obtained from shear tests on single-lap joints: (**a**) unreinforced specimens; (**b**) reinforced specimens.

Figure 11 illustrates the load/displacement curves obtained from experiments.

**Figure 12:**
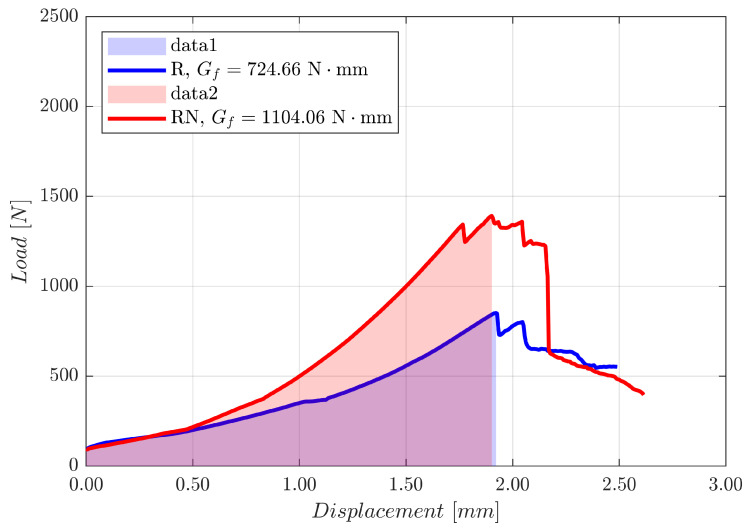
Calculation of experimental fracture energy.

Figure 12 illustrates the calculation of experimental fracture energy.

Table 5 summarises the average mechanical parameters of the tested joints.

**Figure 13:**
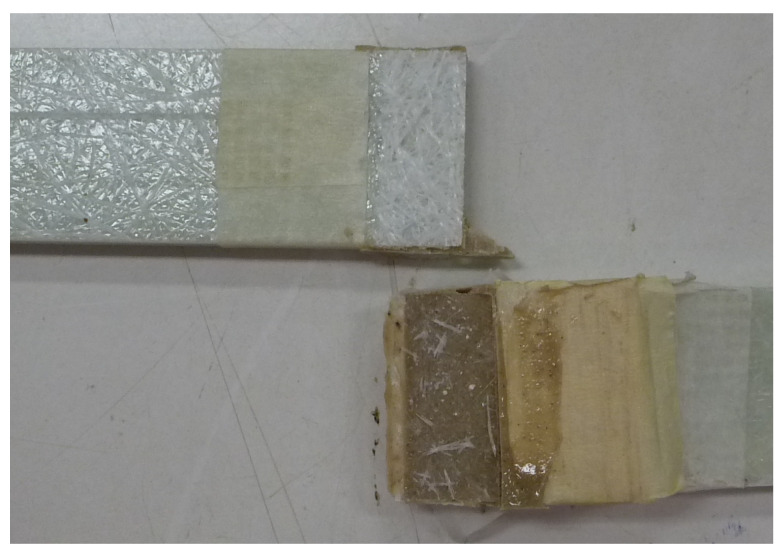
Typical failure LFTF mode of the specimens.

The configuration reinforced with a nylon 6 fabric in the mid-plane of the resin layer showed the best response in terms of stiffness, although it had a lower maximum strength than the unreinforced joint. The introduction of the nylon reinforcement led to an increase in stiffness in both sections of the load/displacement curve, with an increase of +17% in the first (initial) phase and +6% in the second. However, a slight decrease in breaking strength (−8.42%) was observed compared to the configuration without reinforcement. This behaviour illustrates that although the nylon reinforcement does not improve the load-bearing capacity, it helps to make the joint stiffer and more stable under moderate loads, as the equivalent modulus of elasticity of the composite system is increased overall. In practice, the nylon insert in the adhesive alters the mechanical response of the joint: it increases stiffness but slightly impairs ductility and fracture resistance, probably due to the expected initiation of localised damage or disruption of the optimum stress distribution. Figure 13 shows the adhesive joint after the test, highlighting an LFTF failure with minimal and slight removal of the adhering surface fibres, a phenomenon that can be assimilated to an adhesive failure mode.

### 5.4. Comparison of Experimental and Analytical Results

**Figure 14:**
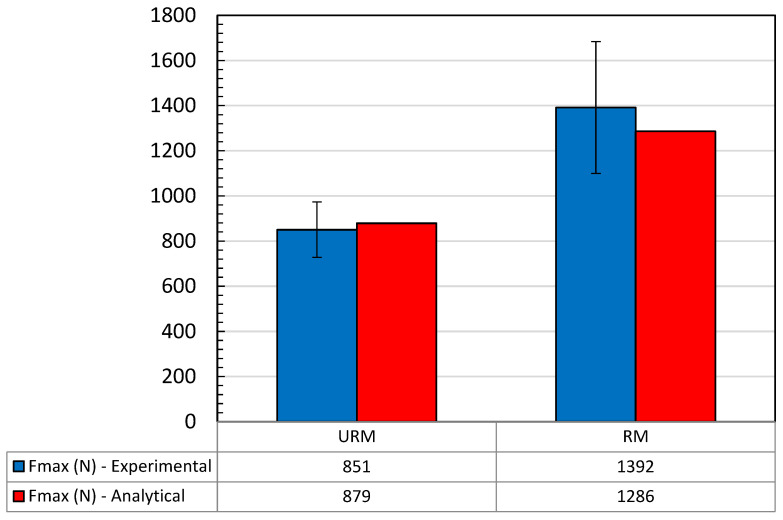
Values of ultimate loads obtained from experimentation by analytical method.

Taking into account the theoretical model and the experimental results, a comparison can be made between the load capacity obtained from the experiments and the load capacity that can be calculated using the relationships (26) and (29). Figure 14 illustrates the values of ultimate loads obtained from experimentation by the analytical method.

The comparison between the results of the experimental campaign and the results of the developed analytical model showed a limited deviation of about 3% for unreinforced joints and −8% for reinforced joints under the conditions without external prestressing. These deviations are within an acceptable range and indicate a good reliability of the theoretical model, especially for reinforced joints where the hypothesis of stress distribution fits better with the actual behaviour. The better agreement in the reinforced joints suggests that the reinforcement introduced (nylon in this case) helps to regulate the stress field and reduce the uncertainties associated with the local variability of the adhesive behaviour, favouring a more predictable response and better described by simplified theoretical models.

These results confirm the validity of the proposed analytical approach, which can be a useful tool for the preliminary design of bonded joints, even when the inner reinforcement is present.

## 6. Discussion

The combined analysis between the analytical model and the experimental data made it possible to highlight the main effects of the insertion of a textile reinforcement in the adhesive layer of single-lap joints and the significant influence of the initial prestressing on the tensile behaviour of the system. Firstly, the good correlation between the theoretical predictions and the experimental results—with small deviations of 3% for unreinforced joints and −8% for reinforced joints—confirms the validity of the chosen modelling approach, which, despite its simplicity, is able to effectively describe the distribution of axial stresses in the adhesives and the reinforcement, as well as the tangential stresses in the adhesive layer.

From a mechanical point of view, the presence of the nylon 6 reinforcement, located in the mid-plane of the adhesive, led to a significant increase in the stiffness of the joint, especially in the initial phase of the reaction, suggesting a greater ability to stabilise under modal loads. This effect leads to a more linear and elastic behaviour in the first part of the load/displacement curve. At the same time, a slight reduction in ultimate strength was observed, due to localisation phenomena of damage at the adhesive interface, limited by the discontinuity introduced by the reinforcement itself. The reinforcement therefore acts as a passive stiffening element that makes the reaction more stable and predictable without increasing the maximum load-bearing capacity of the connection.

A central role was played by the parametric analysis of the initial prestress, which showed significant effects on the overall stress state. The introduction of a distributed prestress in the reinforcement leads to a favourable redistribution of the stresses with a progressive reduction in the axial stresses on the external adhesives and a partial absorption of the load by the reinforcement. In the theoretical models, this leads to a reduction in the stress peaks within the adhesive layer, especially at the ends, which are the critical zones for the onset of debonding. It was observed that with increasing prestress, the localised stresses in the adhesive are reduced and the stress gradient along the overlap is weakened, an effect that is particularly evident in the reinforced configuration.

Furthermore, the analysis has shown that the prestressing acts not only as a stabilising force but also as a “prestressing mechanism” of the joint, which can influence the stress equilibrium already in the first phases of external load application. This leads to a better deformation compatibility between the individual materials and to a more even distribution of internal stresses, especially in connections with flexible but prestressed reinforcement. This effect is consistent with the reduction in shear peaks at the ends of the adhesive layer observed in the numerical results and suggests that the controlled use of prestressing may be a key factor in improving the durability of joints.

Overall, the work has shown that the combination of textile reinforcement and internal prestressing is an effective strategy for optimising the mechanical response of bonded joints by modulating stiffness and improving stress distribution without significantly compromising load-bearing capacity. These results pave the way for future developments in the design of hybrid joints, where reusable flexible materials and prestressing techniques can be combined to customise the response of the system to specific project requirements, especially in lightweight construction sectors such as aerospace, transportation and prefabrication.

## 7. Conclusions

The results obtained in the present work, through an integrated approach between experiment and analytical modelling, allowed an in-depth evaluation of the effects of the insertion of textile reinforcement and distributed prestressing in the adhesive layer of single-lap joints. The main results can be summarised as follows:

- The developed analytical model proved to be effective in describing the stress distribution in adhesive joints with a deviation of 3% for unreinforced joints and −8% for reinforced joints when no external prestressing is present;
- The nylon 6 reinforcement introduced in the adhesive mid-plane of the adhesive resulted in an increase in the joint stiffness, especially in the initial phase, and a slight reduction in the ultimate strength, due to the localisation of the stresses;
- The internal prestressing of the reinforcement led to a reduction in the axial stresses in the outer adherends, to a reduction in the shear peaks in the adhesive layer, especially at the ends, and to a more even distribution of the stresses along the overlap length;
- From a functional point of view, the prestressing served as a mechanism for pretensioning the joint and as an element of deformation compatibility between the materials involved;
- The combination of textile reinforcement and prestressing makes it possible to selectively modulate the mechanical response of the joint and improve stiffness and durability without significantly compromising the load-bearing capacity.

In summary, the results obtained suggest that the joint use of textile materials and prestressing strategies is a promising solution for the performance improvement of adhesive joints, especially in lightweight applications where reliability, stiffness and control of the stress state are important design elements. Further developments could extend the analysis to cyclic loading and critical environmental conditions to assess long-term durability.

## Figures and Tables

**Table 1:** Mechanical and geometrical parameters (refer to Figure 3).

Configuration	E_1_ (MPa)	E_2_ (MPa)	G_a_ (MPa)	b (mm)	t_1_ (mm)	t_2_ (mm)	t_a_ (mm)	l (mm)	L (mm)	P (MPa)	F (MPa)
RM	26,000	110,000	1838	20	5	0.24	2 + 2	12.70	100	20	20

**Table 2:** Mechanical properties according to manufacturer’s data sheet.

GFRP Profiles *		
E_t_ (MPa)	σ_t_ (MPa)	ε_t_ (%)
26,000	400	1.50

* according to EN 755-2 [37].

**Table 3:** Mechanical parameters of the nylon 6 reinforcement as reported by manufacturer.

Nylon 6	
Type	A
Dtex at brill (kg/base)	0.840/96
Wires	5600
Mechanical stop	7.50
Fabric armour	tela
Weight (kg/m)	0.0335
Thickness (mm)	0.24
Elastic modulus in tension (MPa)	2600

**Table 4:** Technical and mechanical properties of the adhesive reported by manufacturers.

EPX-RN	
Chemical base	Two-part epoxy
Viscosity	Thixotropic Paste
W_t_ (min)	15–45
A_t_ (°C)	>5
S_t_ (°C)	10–35
τ (MPa)	6
σ_t_ (MPa)	5.50
E_t_ (MPa)	5000
Use	Structural

**Table 5:** Average mechanical parameters of the tested joints.

Specimen	Nylon Reinforcement	F_max_ (N)	s (mm)	G_f_ (Nmm)
URM	-	850.74 ± 245	1.92 ± 1.10	724.66
RM	Yes	1391.57 ± 584	1.90 ± 0.20	1104.06

## Data Availability

The original contributions presented in this study are included in the article. Further inquiries can be directed to the corresponding author.
